# Playing the Field: Sox10 Recruits Different Partners to Drive Central and Peripheral Myelination

**DOI:** 10.1371/journal.pgen.1003918

**Published:** 2013-10-31

**Authors:** Ben Emery

**Affiliations:** Department of Anatomy and Neuroscience and Florey Institute of Neuroscience and Mental Health, University of Melbourne, Melbourne, Australia; Stanford University School of Medicine, United States of America

Within the vertebrate nervous system, specialized glial cell types ensheath the axons of neurons with multiple wraps of membrane (myelin) in order to increase the speed and efficiency of nerve conduction. In the central nervous system, this role is fulfilled by oligodendrocytes; Schwann cells carry out the equivalent function within the peripheral nervous system. In spite of their common function, there are some substantial differences between oligodendrocytes and Schwann cells. For starters, they have different embryonic origins, arising from the neural tube and neural crest respectively. Each oligodendrocyte may myelinate anywhere from 1 to 50 axons, whereas a myelinating Schwann cell will devote its energy to a single axon. Even the major protein components of myelin in the peripheral and central nervous systems are a somewhat inexplicable mix; both incorporate the Myelin Basic Protein (MBP), but the major peripheral myelin protein, Protein Zero (P_0_), is replaced in the central nervous system with Proteolipid Protein (PLP). The transcription networks underlying differentiation and myelination in each cell type are also largely distinct, although one consistency is that the HMG-domain transcription factor Sox10 is required for successful myelination by both cell types [1], [2].

In this issue of *PLOS Genetics*, Hornig and colleagues [3] give another striking example of two very different genes being co-opted by Sox10 to drive the myelination process in each cell type.

**Figure 1:**
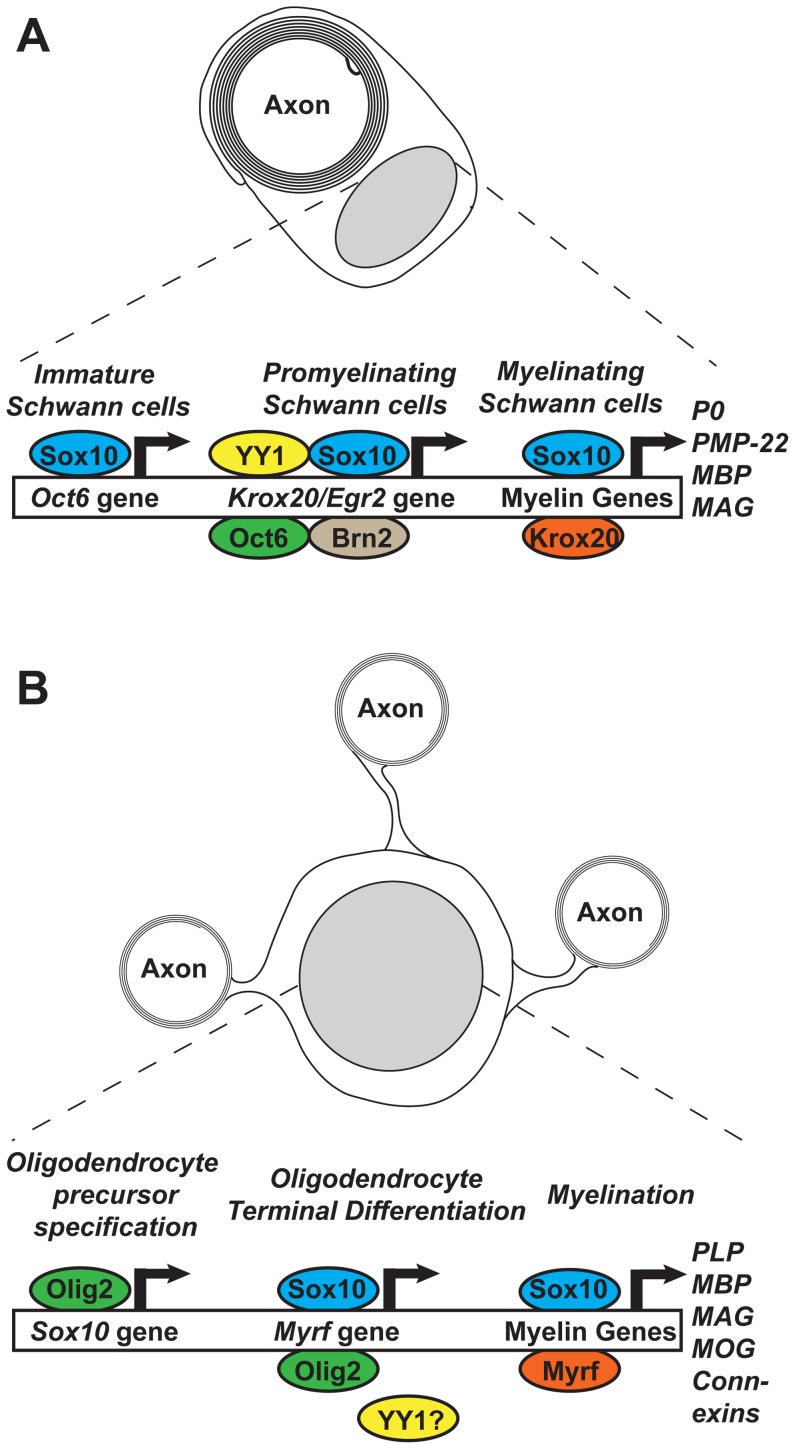
Feed-forward transcriptional networks regulating myelination in Schwann cells and oligodendrocytes. (A) Sox10 is present within immature Schwann cells and acts to induce expression from the *Oct6* gene via a downstream enhancer [13]. Upon signaling from axonal neuregulin, Sox10, Oct6, Brn2, and YY1 act to drive Krox20 expression via a +35 kb upstream enhancer [4], [14]. Krox20 and Sox10 then synergistically activate myelin genes. (B) Within the developing central nervous system, Olig2 acts during oligodendrocyte precursor specification to induce the expression of Sox10 [15]. During terminal differentiation, Sox10 and Olig2 induce the expression of Myrf, with Sox10 binding the intron 1 enhancer/ECR9 [3]. Sox10 and Myrf then act at myelin gene promoters and enhancers to drive myelin gene expression. See [16], [17] for a more comprehensive review of the mechanisms controlling Schwann cell and oligodendrocyte development and myelination, respectively.

Within the Schwann cells, Sox10 is known to directly induce the transcription of another transcription factor, Krox20 (also known as Egr2) [4]. Sox10 and Krox20 subsequently act in concert at myelin gene enhancers [5], [6] (Figure 1). Unlike Schwann cells, oligodendrocytes do not express Krox20, however recent work has identified a putative functional replacement, Myelin Regulatory Factor (Myrf, previously known as C11Orf9, MRF, and GM98). Just as Krox20 is upregulated in myelinating Schwann cells, Myrf is upregulated during oligodendrocyte differentiation and is required for them to myelinate [7].

Hornig and colleagues now demonstrate that Krox20 and Myrf not only have an analogous role in driving myelination in their respective cell types, they also share a remarkably similar relationship with Sox10. Just as Sox10 directly regulates Krox20 in Schwann cells, they find it is also required for the induction of Myrf during terminal oligodendrocyte differentiation in vivo. This regulation by Sox10 was mapped to an enhancer in the first intron of the *Myrf* gene containing several Sox consensus motifs. They show this enhancer is bound by Sox10 and is sufficient to drive gene expression in developing oligodendrocytes.

Perhaps equally strikingly, the Sox10 and Myrf proteins were found to subsequently physically interact and act synergistically at key myelin gene enhancers, including upstream of the MBP gene. This corroborates recent ChIP-Seq data indicating that the two bind to partially overlapping genomic regions within oligodendrocytes [8]. This, once again, closely mirrors the functional relationship between Sox10 and Krox20 in the PNS, where they act synergistically in the myelinating Schwann cells to regulate myelin gene expression [5], [6]. Intriguingly, both Hornig et al. and Bujalka et al. find that although there is some clear overlap and synergy between the myelin gene regulatory regions bound by Sox10 and Myrf, there are also some distinct differences, with many regulatory elements being targeted by just one factor [3], [8]. This suggests that the two do not necessarily act as part of an obligatory protein complex, instead sharing overlapping but subtly distinct roles.

These findings further cement the central role of Sox10 in the regulation of the differentiation of both Schwann cells and oligodendrocytes and their subsequent myelination. Indeed, its role is remarkably well-conserved in peripheral and central glia given how few other key transcription factors are common between the two. As Hornig et al. point out, on the surface Myrf appears to be an unlikely functional replacement for Krox20. Krox20 is a fairly well characterized zinc finger transcription factor, with a variety of roles in development. In contrast, Myrf is something of the eccentric elderly uncle in the transcription factor family. It has few close homologs but shows homology to the yeast transcription factor Ndt80 [9], also incorporating structural domains from bacteriophage proteins [8], [10]. Within vertebrates, its roles outside central nervous system myelination remain undefined. Nevertheless, the parallels between Krox20 and Myrf, as well as the relationship they share with Sox10, are clear.

A number of questions are raised by these findings.

As Hornig et al. note, Sox10 is present within the oligodendrocyte precursors for some time before it acts to promote the expression of Myrf. This indicates the presence of additional regulatory mechanisms. Recent work suggests that chromatin remodeling by Brg1 and Olig2 alters the accessibility of key genes, including *Myrf*, at the onset of terminal differentiation [11]. It is highly feasible that other factors such as Nkx2.2 and YY1 will also have a direct role in this regulation. The broader cellular events and molecular partners that direct Sox10 to the *Myrf* intronic enhancer at the critical point of oligodendrocyte differentiation will be important to determine.

Secondly, Hornig and colleagues found that Sox10 physically interacts with the C-terminal region of Myrf. Several groups have recently reported that the Myrf protein is cleaved as a prerequisite for its transcription factor function and that the C-terminal domain appears to be excluded from the nucleus [8], [10], [12]. The functional role of this physical interaction between Sox10 and the C-terminal of Myrf (and indeed the role of the C-terminal domain of Myrf more generally) will therefore be important to clarify.

Finally, and perhaps most intriguingly, the question of how Schwann cells and oligodendrocytes have come to perform essentially the same function (ensheathing nerve cells) during vertebrate evolution while using overlapping, but clearly discrete, sets of transcription regulators remains to be resolved. Do they share a common cellular ancestor, or (as Hornig and colleagues speculate), has the myelination process evolved independently in the central and peripheral nervous systems with Sox10, the common element in an otherwise largely mixed bag of genes? It seems possible that careful analysis of the neural cells that co-express Sox10, Krox20, and Myrf in our evolutionary distant relatives may hold the key to the origins of these cells and their relationship to each other.
